# Identification of Hub Genes and Immune-Related Pathways for Membranous Nephropathy by Bioinformatics Analysis

**DOI:** 10.3389/fphys.2022.914382

**Published:** 2022-06-24

**Authors:** Xiao-Yu Cai, Zu-Feng Wang, Shu-Wang Ge, Gang Xu

**Affiliations:** Division of Internal Medicine, Department of Nephrology, Tongji Hospital, Tongji Medical College, Huazhong University of Science and Technology, Wuhan, China

**Keywords:** bioinformatics analysis, membranous nephropathy, hub genes, immunology, GSEA

## Abstract

**OBJECTIVE:** We aim to explore the detailed molecular mechanisms of membrane nephropathy (MN) related genes by bioinformatics analysis.

**METHODS:** Two microarray datasets (GSE108109 and GSE104948) with glomerular gene expression data from 65 MN patients and 9 healthy donors were obtained from the Gene Expression Omnibus (GEO) database. After processing the raw data, DEGs screening was conducted using the LIMMA (linear model for microarray data) package and Gene set enrichment analysis (GSEA) was performed with GSEA software (v. 3.0), followed by gene ontology (GO) enrichment and Kyoto Encyclopedia of Genes and Genomes (KEGG) pathway enrichment. The protein-protein interaction (PPI) network analysis was carried out to determine the hub genes, by applying the maximal clique centrality (MCC) method, which was visualized by Cytoscape. Finally, utilizing the Nephroseq v5 online platform, we analyzed subgroups associated with hub genes. The findings were further validated by immunohistochemistry (IHC) staining in renal tissues from MN or control patients.

**RESULTS:** A sum of 370 DEGs (188 up-regulated genes, 182 down-regulated genes) and 20 hub genes were ascertained. GO and KEGG enrichment analysis demonstrated that DEGs of MN were preponderantly associated with cell damage and complement cascade-related immune responses. Combined with literature data and hub gene-related MN subset analysis, CTSS, ITGB2, and HCK may play important roles in the pathological process of MN.

**CONCLUSION:** This study identified novel hub genes in MN using bioinformatics. We found that some hub genes such as CTSS, ITGB2, and HCK might contribute to MN immunopathological process, providing new insights for further study of the molecular mechanisms underlying glomerular injury of MN.

## Introduction

Membrane nephropathy (MN) is an autoimmune illness defined by the development of typical glomerular deposits containing immunoglobulin and complement components (Couser, 2017). Globally, the incidence of MN is around 1/100,000, with about 80% being primary membranous nephropathy (PMN) (McGrogan et al., 2011). Clinically, the therapeutic outcome of MN is not promising, with recurrent MN occurring in approximately 25%–30% of cases in complete remission (Polanco et al., 2010). Moreover, 20%–30% of patients with MN eventually develop end-stage renal disease (Trujillo et al., 2020).

Pathologically, MN is characterized by thickening of the glomerular basement membrane (GBM) and extensive disappearance of foot processes (Fogo et al., 2015). Several significant target antigens have been found in MN, including the phospholipase A2 receptor (PLA2R), thrombospondin type 1 domain-containing 7A (THSD7A), neural epidermal growth factor-like 1 (Nell-1), and semaphorin-3B (Sema3B) (Cattran and Brenchley, 2017; Sethi et al., 2020a; Sethi et al., 2020b), with more under investigation. During the pathogenesis of MN, the immune tolerance of these proteins is broken, activating the immune pathway to produce antibodies against these podocyte target antigens, resulting in the production and aggregation of subepithelial immune deposits. Simultaneously, IgG may be involved in activating the lectin pathway of complement or the classical pathway, triggering a complement cascade process that results in the production of the podocyte lesion membrane assault complex C5b-9 (Borza, 2016). With an increasing number of therapeutic agents available to target the complement activation pathway, it will be crucial to understand the precise function of complement in the development and maintenance of MN. Moreover, identifying biomarkers and potential molecular mechanisms associated with pathological alterations in MN requires further research, which will lead to the development of novel diagnostic and treatment strategies for MN.

The aim of this study was to explore the detailed molecular mechanism of MN-related genes. With microarray data obtained from the GEO database, we first evaluated two microarray datasets of glomerular gene expression data from 65 patients with MN and 9 healthy donors. DEGs screening was performed between MN and the control group after raw data processing. Possible pathogenesis of MN was then explored by employing gene ontology (GO) enrichment, Kyoto Encyclopedia of Genes and Genomes (KEGG) enrichment, and network analysis of protein-protein interaction (PPI). Gene set enrichment analysis (GSEA) was conducted with GSEA software (v. 3.0). Finally, we adopted the Nephroseq V5 online platform to complete the hub gene-related subgroup analysis. In conclusion, we identified 370 DEGs and 10 hub genes that may serve as diagnostic indicators and therapeutic targets for prevention of MN occurrence and development.

## Materials and methods

### Microarray Datasets Acquisition

We obtained the required microarray data from the GEO database. GEO (Barrett et al., 2013) is the National Center for Biotechnology Information’s public functional genomic data repository, which has a massive collection of high-throughput gene expression data, chips, and microarrays. Using “membranous nephropathy” as the searching keyword, potential GEO datasets were selected according to the following inclusion criteria: 1) The research type was array-based expression profiling; 2) The organisms studied had to be *Homo sapiens*; 3) Each dataset’s sample included glomerular tissue; 4) The total sample size was greater than 15. According to the above criteria, we chose the membranous nephropathy samples in microarray dataset GSE108109 (Grayson et al., 2018) based on GPL19983 (Affymetrix Human Gene 2.1 ST Array) platform, and GSE104948 (Grayson et al., 2018) based on GPL22945 (Affymetrix Human Genome U133 Plus 2.0 Array) and GPL24120 (Affymetrix Human Genome U133A Array) for further analysis. GSE108109 collected 44MN samples of glomerular tissue and 6 healthy controls, whereas GSE104948 collected 21MN samples and 3 healthy controls. We downloaded a series of matrix files and data table descriptions to screen and validate key genes associated with glomerular lesions in MN. The sample data for this research were freely available from public sources and therefore did not require patient consent or ethics committee approval.

### Data Preprocessing and Differentially Expressed Genes Identification

Preprocessing of data comprised converting probes to gene symbols, integrating data sets, and batch normalization. The mRNA probes were annotated using annotation files. The probes not mapping to any genes were discarded, whereas numerous probes that match to the same mRNA were averaged. Affymetrix platform’s original matrix data was read by AFFY package (Gautier et al., 2004) of R software (version 3.5.2), and background correction and normalization were performed by robust multi-array averaging (RMA) algorithm (Bolstad et al., 2003). The combined data was then preprocessed using the SVA package (Leek et al., 2012) to eliminate the batch effect. To test for differentially expressed genes across renal glomerular tissues from MN and healthy controls, we utilized the LIMMA (linear model of microarray data) (Smyth, 2004) package of R program. The adjusted *p* value of 0.05 was used as the threshold, as was |log FC| (fold change) > 1. All significant DEGs were shown on the volcano map generated using the R software. The heatmap of DEGs was drawn with Pheatmap package.

### Gene Ontology and Functional Enrichment Analyses

DAVID 6.8 (database for annotation, visualization, and comprehensive discovery) (Huang et al., 2007) is an online bioinformatics database that provides functional annotation tools comprehensively for elucidating the biological significance of target genes and molecular pathways by combining biological data and analytical techniques. Functional enrichment study on DEGs was performed utilizing DAVID, including functional classification, gene ontology (GO) terminology, and the Kyoto Encyclopedia of Genes and Genomes (KEGG) pathway. GO (Gene Ontology, 2015) analysis is a frequently used and beneficial technique in large-scale functional enrichment studies. It is used to identify specific biological properties and assign protein biomarkers to corresponding pathways at the levels of biological process (BP), cell composition (CC) and molecular function (MF). KEGG (Wixon and Kell, 2000) pathway enrichment analysis was used to allocate multiple sets of DEGs to distinct pathways for determining functional attributes. The threshold values were gene count >2 and *p* < 0.05. Gene Set Enrichment Analysis (GSEA) (Subramanian et al., 2005) is a statistical method for analyzing whole-genome expression profile microarray data. It can sequence genes between two samples based on the degree of differential expression, and the enrichment order of pre-defined genomes in sequencing tables was assessed subsequently to determine whether the expression of genes from a particular pathway or other predefined gene set between two groups show statistically significant difference. In this study, GSEA software (version 3.0) was utilized to perform GO analysis of all detected genes.

### Protein-Protein Interaction Network Construction and Hub Gene Identification

To identify and assess protein functional relationships and protein-protein interaction (PPI) networks for differentially expressed mRNAs, we utilized the Search Tool for the Retrieval of Interacting Genes (STRING 10.5) (Szklarczyk et al., 2015). These interactions included both physical and functional relationships and were based on data from automated text mining, high-throughput tests, and co-expression networks. We linked all acquired DEGs to the String database and the interaction score threshold was fixed to >0.7 to represent high confidence interactions. The results of the STRING analysis were then imported into Cytoscape (Shannon et al., 2003), which was used to select the key nodes with the strongest connectivity to visualize the molecular interaction network. The nodes with the most interactions with neighboring nodes were considered as the key node. To look for clusters and key genes in highly interconnected regions of the PPI network, Clustering analysis of differential genes was then applied using the Molecular Complex Detection (MCODE 1.5.1) plug-in for the Cytoscape software (Bandettini et al., 2012), whose purpose is to cluster networks based on topology to identify areas that are closely connected. DEGs clustering and scoring parameters were set as follows: MCODE Score ≧4, degree cutoff = 2, node cutoff = 0.2, maximum depth = 100, K-score = 2.

### Nephroseq v5 Validation

Nephroseq V5 (http://v5.nephroseq.org) (Eddy et al., 2020) is a non-profit, integrated data mining platform for comprehensive kidney disease gene expression datasets. To verify the robustness of our results in the external dataset, the expression levels of key genes associated with MN were compared with normal controls using the Nephroseq V5 online database. Statistical significance was defined as *p* value less than 0.05.

### Immunohistochemical Staining

Formalin fixed paraffin-embedded renal tissue were derived from our pathology files. Kidney tissue of membranous nephropathy was from kidney puncture and control group was non-tumor tissue adjacent to renal tumor. Each tissue block was cut into 4 μm slices, baked at 70°C for 1 h, soaked in xylene and anhydrous ethanol for 30 min respectively, and then treated with adding citrate buffer (pH 6.0) 20 min for antigen retrieval. Subsequently, the section was sealed for 15 min at room temperature in 10% hydrogen peroxide endogenous peroxidase, blocked 30 min with 5% secondary antiserum, and incubated overnight at 4°C with anti-CTSS (sc-271619, anta Cruz Biotechnology), HCK (sc-8420, anta Cruz Biotechnology), ITGB2 (sc-101428, anta Cruz Biotechnology) first antibody and treated with horseradish peroxidase (HRP)-labeled secondary antibody. Following three rinses with PBS, the slices were dyed with 3,3′-diaminobenzidine (DAB). Representative photos were captured using an Olympus microscope equipped with a DP73 digital camera.

## Result

### Identification of Data Preprocessing and Differentially Expressed Genes in Membrane Nephropathy Using Integrated Bioinformatics

**FIGURE 1:**
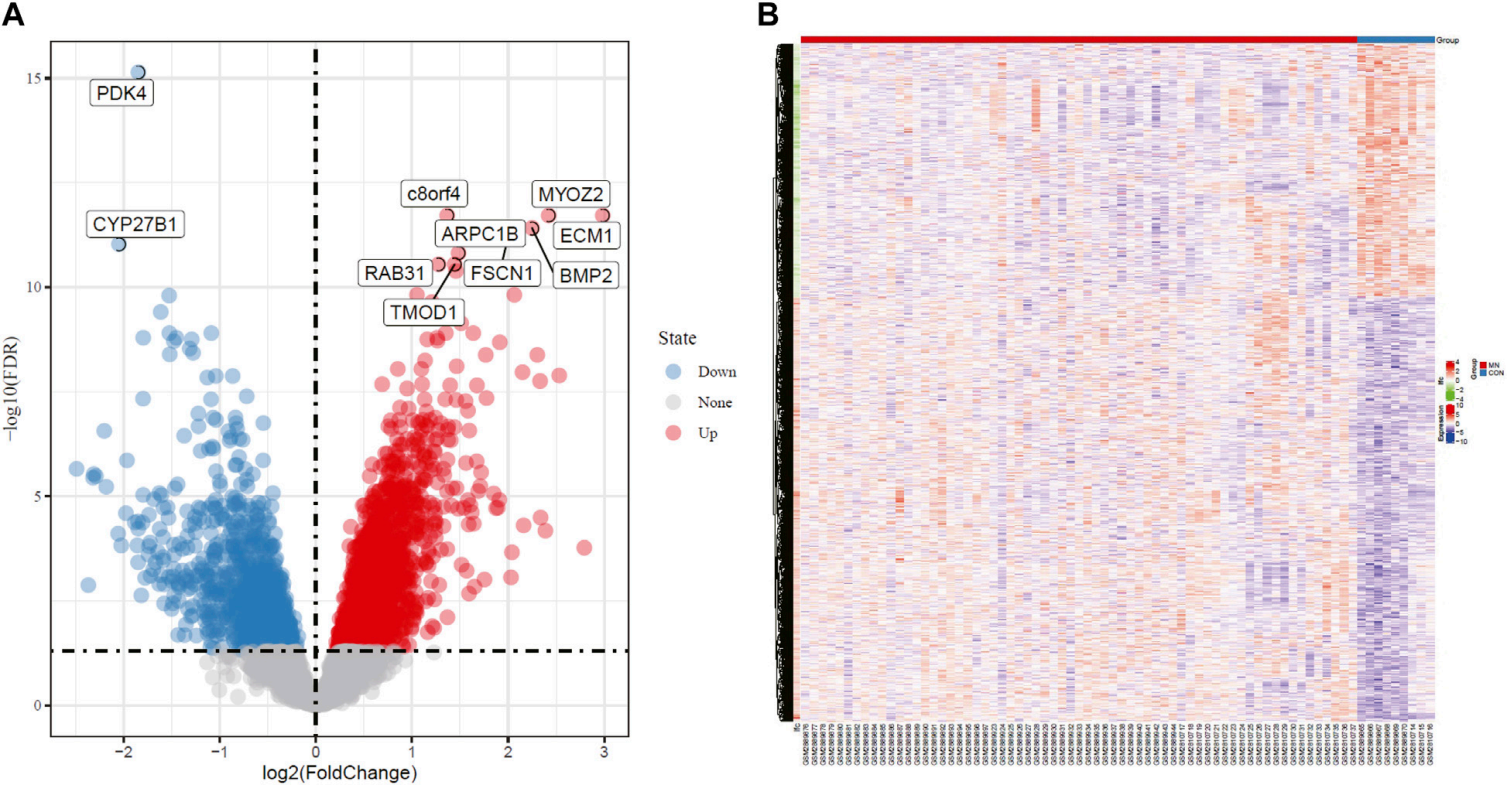
Volcano plot and Heatmap analysis identifies DEGs. **(A)** Red dots represent upregulated genes and blue dots represent downregulated genes in renal glomerular tissue from MN patients compared with normal controls. **(B)** Red areas represent highly expressed genes and blue areas represent lowly expressed genes in renal glomerular tissue from MN patients compared with normal controls. DEG, differentially expressed gene; MN, membranous nephropathy.

Two independent data sets (GSE108109 and GSE104948) were retrieved from GEO. 9 glomerular samples from normal functioning individuals and 65 glomerular samples from MN patients were selected for analysis. The original matrix data was normalized by RMA method from LIMMA package, after which differential expression analysis of these data sets was conducted (adjust *p* value <0.05, | logFC | > 1). According to the sample information and gene expression matrix, 370 DEGs were screened, of which 188 were up-regulated genes and 182 were down-regulated genes, as shown in the volcano map (Figure 1A). In the heat map (Figure 1B), the horizontal axis represented the included samples (MN or healthy control group), the vertical axis represented the differentially expressed genes, and the color represented the relative gene expression level, with red being high expression and blue being low expression. The contrast between differential gene expression in the MN and control groups was remarkable, where a portion of genes that were lowly expressed in the control group were highly expressed in the MN group, while another portion of genes that were highly expressed in the control group were lowly expressed in the MN group. Table 1 and table 2 demonstrate the top 10 up-regulated and down-regulated DEGs.

**TABLE 1:** Top 10 up-regulation differentially expressed genes in MN group.

Rank	Gene symbol	Gene description	logFC	Adj.*p.*val
1	ECM1	Extracellular matrix protein 1; interacts with a variety of extracellular and structural proteins	2.99	<0.001
2	COLEC12	Collectin subfamily member 12; a member of the C-lectin family, proteins that possess collagen-like sequences	2.79	<0.001
3	FCER1G	Fc fragment of IgE receptor Ig; involved in allergic reactions	2.53	<0.001
4	MYOZ2	Myozenin 2; a member of sarcomeric proteins that bind to calcineurin	2.42	<0.001
5	CHGA	Chromogranin A; a member of the chromogranin/secretogranin family of neuroendocrine secretory proteins	2.39	<0.001
6	TGFBI	Transforming growth factor beta induced; encodes an RGD-containing protein that binds to type I, II and IV collagens	2.34	<0.001
7	HBB	Hemoglobin subunit beta; a type of polypeptide chains in adult hemoglobin	2.33	<0.001
8	TNFRSF12A	TNF receptor superfamily member 12A; contributes to endothelial dysfunction	2.30	<0.001
9	BMP2	Bone morphogenetic protein 2; encodes a secreted ligand of the transforming growth factor-beta superfamily of proteins	2.25	<0.001
10	LYZ	Lysozyme; encodes human lysozyme, whose natural substrate is the bacterial cell wall peptidoglycan	2.16	<0.001

**TABLE 2:** Top 10 down-regulation differentially expressed genes in MN group.

Rank	Gene symbol	Gene description	logFC	Adj.*p.*val
1	G6PC	Glucose-6-phosphatase; a multisubunit integral membrane protein of the endoplasmic reticulum	−2.49	<0.001
2	CALB1	Calbindin 1; a member of the calcium-binding protein superfamily that includes calmodulin and troponin C	−2.37	<0.001
3	SLC22A8	Solute carrier family 22 member 8; involved in the excretion of some toxic organic anions	−2.32	<0.001
4	ALB	Albumin; encodes the most abundant protein in human blood	−2.31	<0.001
5	AFM	Afamin; a member of the albumin gene family	−2.29	<0.001
6	PCK1	Phosphoenolpyruvate carboxykinase 1; a main control point for the regulation of gluconeogenesis	−2.20	<0.001
7	CYP2B6	Cytochrome P450 family 2 subfamily B member 6; a member of the cytochrome P450 superfamily of enzymes	−2.18	<0.001
8	PLG	Plasminogen; plays a role in the degradation of extracellular matrices, cell migration, inflammation	−2.06	<0.001
9	CYP27B1	Cytochrome P450 family 27 subfamily B member 1; a member of the cytochrome P450 superfamily of enzymes	−2.05	<0.001
10	SLC27A2	Solute carrier family 27 member 2; play a key role in lipid biosynthesis and fatty acid degradation	−2.02	<0.001

### Gene Ontology Functional Enrichment Analysis of Data Preprocessing and Differentially Expressed Genes

**FIGURE 2:**
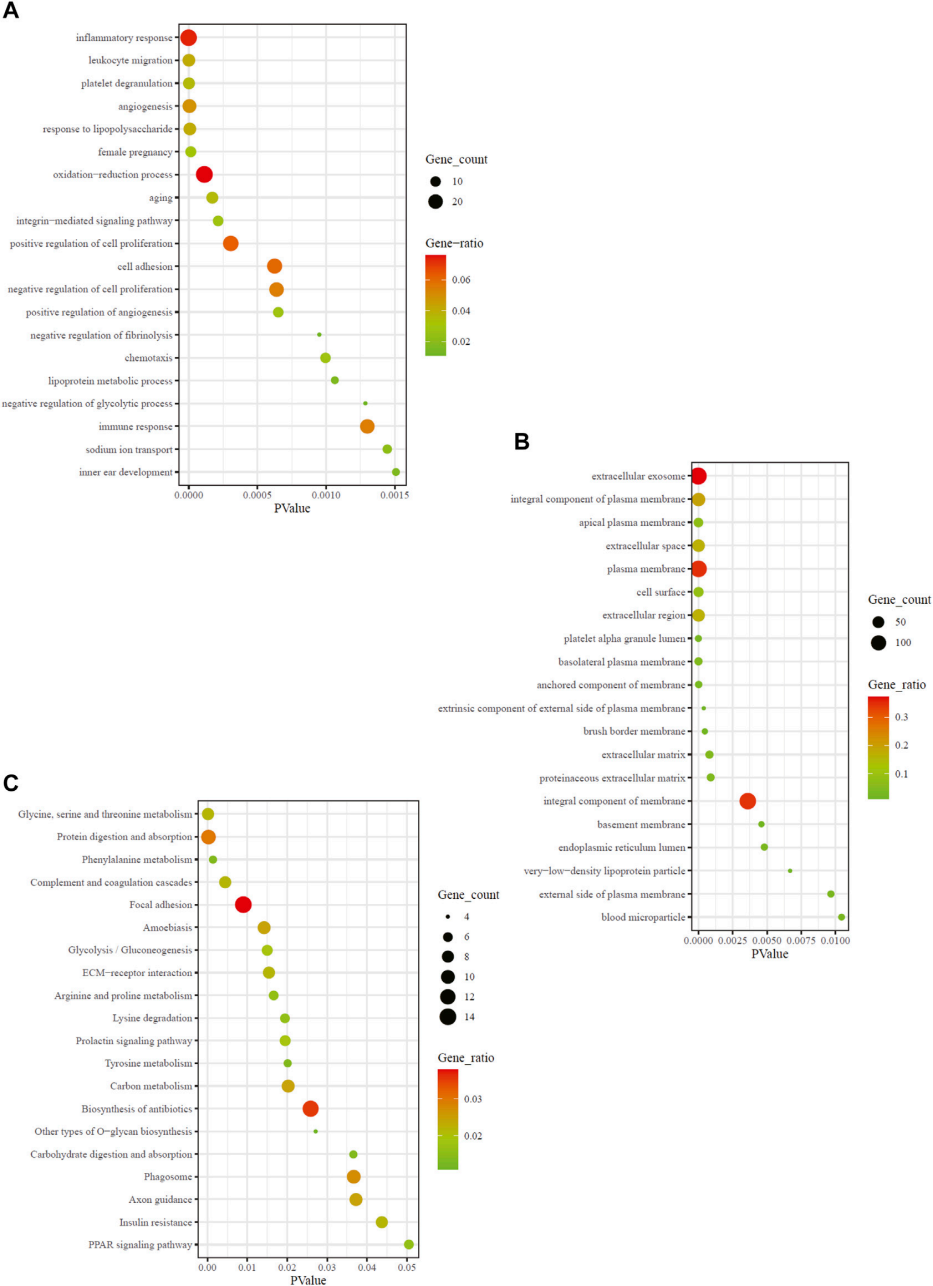
GO and KEGG enrichment result of DEGs. The *x*-axis represents *p* value and *y*-axis represents GO terms. The size of circle represents gene count. Different color of circles represents gene ratio. **(A)** GO-BP enrichment result of DEGs. **(B)** GO-CC enrichment result of DEGs. **(C)** KEGG enrichment result of DEGs. DEG: differentially expressed gene; KEGG: Kyoto Encyclopedia of Genes and Genomes; GO: Gene Ontology; BP: biological process; CC: cell composition.

To ascertain the biological properties of DEGs, we performed GO enrichment analysis on 370 DEGs using David online tools. The top 20 biological processes were screened according to *p* < 0.05, and the bubble plots were drawn according to enrichment fraction. Figure 2A showed that the biological process of significant enrichment was mainly related to angiogenesis and inflammatory immune response. The top 10 BP terms with significant enrichment were “inflammatory response,” “leukocyte migration,” “platelet degranulation,” “angiogenesis,” “response to lipopolysaccharide,” “female pregnancy,” “oxidation-reduction process,” “aging,” “integrin-mediated signaling pathway,” “positive regulation of cell proliferation” respectively. In addition, the top 10 CC terms are selected (Figure 2B), consisting of “extracellular exosome,” “integral component of plasma membrane,” “apical plasma membrane,” “extracellular space,” “plasma membrane,” “cell surface,” “extracellular region,” “platelet alpha granule lumen,” “basolateral plasma membrane,” “anchored component of membrane”. These pathways are critical for elucidating the role of hub genes in MN pathogenesis.

### Kyoto Encyclopedia of Genes and Genomes Pathway Enrichment Analysis of Data Preprocessing and Differentially Expressed Genes

To explore the enrichment pathway of DEGs, David Online Tools were applied to analyze the KEGG pathway. With *p* < 0.05 as the threshold, a total of 20 significant enrichment pathways were screened out (Figure 2C), including “glyeine, serine and threonine metabolism,” “protein digestion and absorption,” “complement and coagulation cascades,” “focal adhesion,” “amoebiasis,” “glycolysis/gluconeogenesis,” “ECM-receptor interaction,” “arginine and proline metabolism,” “lysine degradation.” The findings showed that the hub genes might be involved in MN pathogenesis *via* regulation of amino acid metabolism, complement cascade and inflammatory response. Among them, amino acid metabolic pathways were the most abundant.

### Evaluation of Gene Set Enrichment Analysis-Based Gene Ontology and Kyoto Encyclopedia of Genes and Genomes in Membrane Nephropathy

**FIGURE 3:**
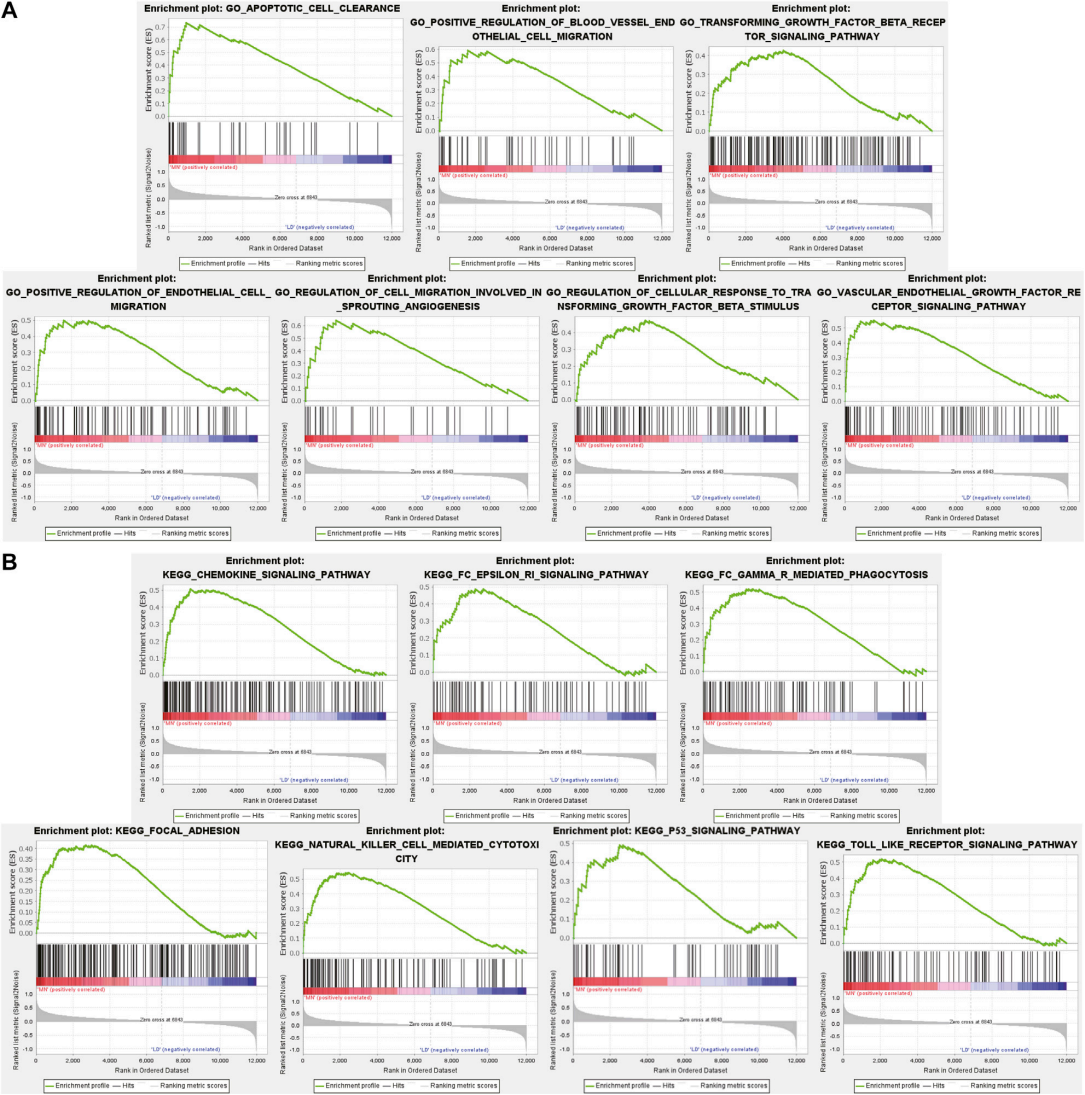
GSEA plot showing most enriched GO terms and KEGG pathways in the MN group. **(A)**. The 7 most significant enriched GO term positively correlated with the MN group was apoptotic cell clearance, positive regulation of blood vessel endothelial cell migration, transforming growth factor beta receptor signaling pathway, positive regulation of endothelial cell migration, regulation of cell migration involved in sprouting angiogenesis, regulation of cellular response to transforming growth factor beta stimulus, vascular endothelial growth factor receptor signaling pathway. **(B)** The 7 most significant enriched KEGG pathway positively correlated with the MN group was chemokine signaling pathway, FcεRi signaling pathway, FcγR mediated phagocytosis, focal adhesion, natural killer cell mediated cytotoxicity, p53 signaling pathway, toll like receptor signaling pathway. MN, membranous nephropathy; GSEA, GO term enrichment analysis; GO, gene ontology; KEGG, Kyoto Encyclopedia of Genes and Genomes.

Different from GO and KEGG analysis, GSEA does not require differential gene threshold. It simply examines whether genes in a collection are randomly distributed or abnormally regulated in certain phenotypes and determines biological associations. Therefore, we applied GSEA to screen for biological differences in glomerular tissue between MN and healthy samples. Figure 3A exhibited the 7 GO-BP terms that were considerably enriched. Compared to the control group, “apoptotic cell clearance,” “positive regulation of blood vessel endthelial cell migration,” “transforming growth factor beta receptor signaling pathway,” “positive regulation of endthelium cell migration,” “regulation of cell migration involved in sprouting angiogenesis,” “regulation of cellular response to transforming growth factor beta stimulus “and “vascular endothelial growth factor receptor signaling pathway” were significantly enriched in MN. These findings underscored the critical importance of apoptosis and immunological modulation in membranous nephropathy. A total of 7 prominent KEGG pathways were selected (Figure 3B), including “Chemokine signaling pathway,” “fcεri signaling pathway,” “fcγr mediated phagocytosis,” “focal adhesion,” “natural killer cell mediated cytotoxicity,” “p53 signaling pathway” and “toll-like receptor signaling pathway,” which were associated with cell damage, release of various inflammatory mediators and cellular immunity. The results showed that the activation of signaling pathways occurred similarly in MN as it did during pathogen infection.

### Protein-Protein Interaction Network Construction and Hub Gene Recognition

**FIGURE 4:**
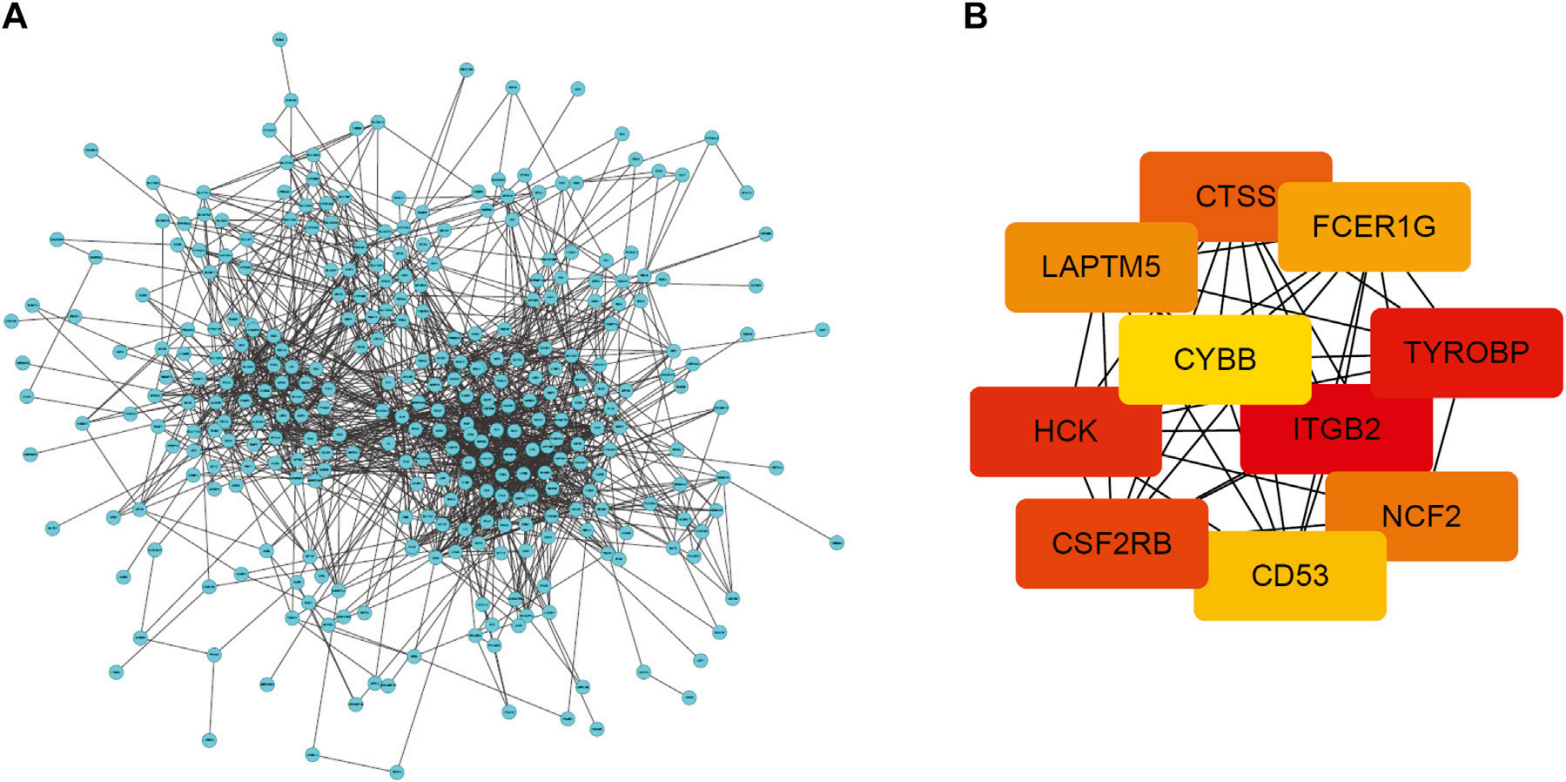
PPI network of DEGs and top 10 hub genes. **(A)** PPI network of DEGs created by STRING. Circles represent genes and lines represent PPIs **(B)** The network of the top 10 hub genes identified by MCODE. DEG: differentially expressed gene; PPI: protein–protein interaction.

To conduct a comprehensive examination of the biological function of DEGs, we performed PPI analysis by the String online database. The interactive network was constructed around the target that had a confidence score of 0.7. After removing orphaned nodes and partially connected nodes, Cytoscape software was used for visualization. Nodes corresponded to genes, and edges represented connections between genes. Shown in Figure 4A, the PPI network had 361 nodes and 1,461 edges. Then, using the MCODE plugin, we screened the clusters within the PPI network and selected the ten genes with the highest score as hub genes (Figure 4B).

### Validations of Association Between Hub Genes and Membrane Nephropathy

**FIGURE 5:**
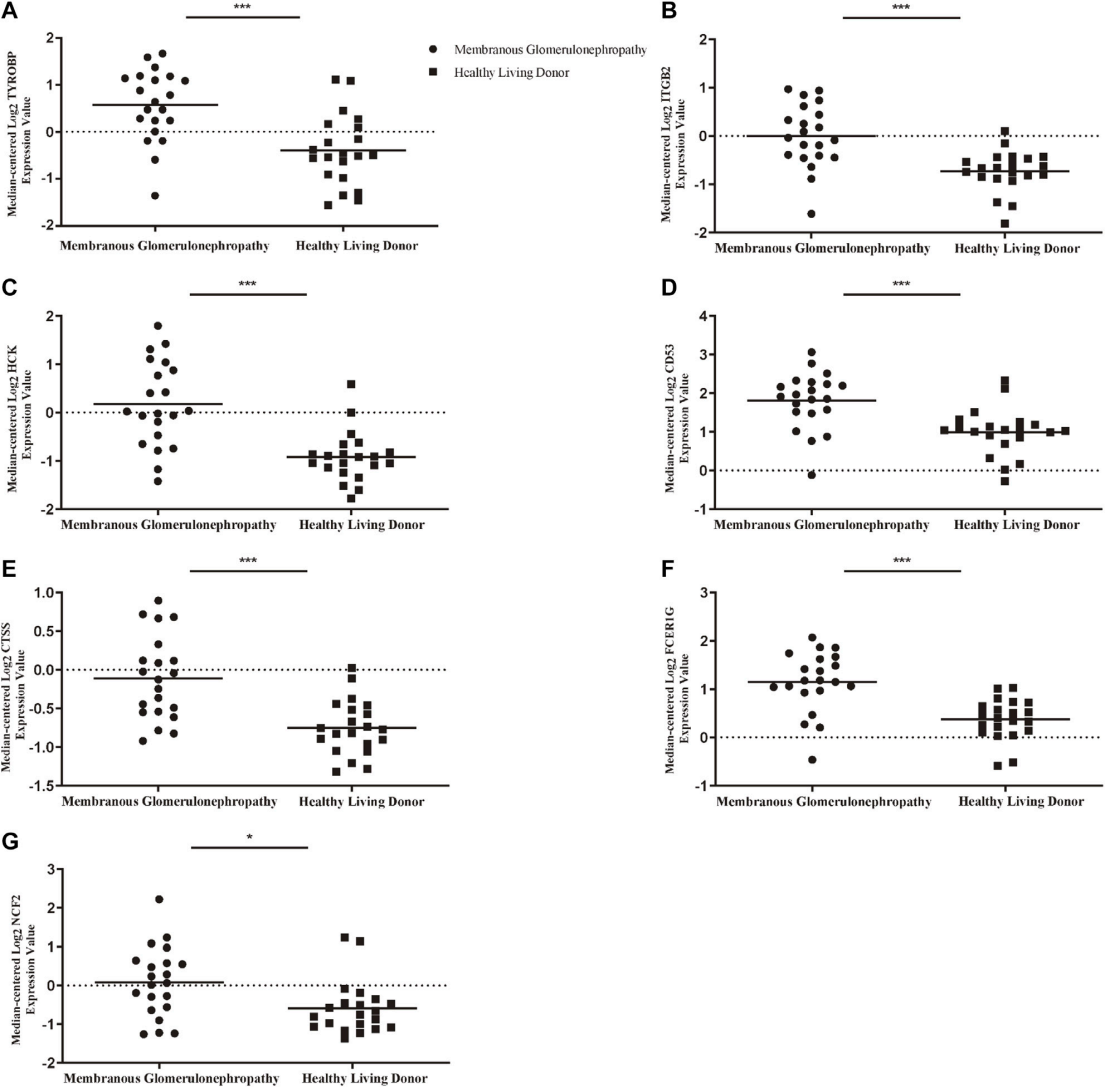
Association between mRNA expression of hub genes in MN patients and normal controls. **(A)** The expression of TYROBP in MN group was higher than that of normal control **(B)** The expression of ITGB2 in MN group was higher than that of normal control. **(C)** The expression of HCK in MN group was higher than that of normal control **(D)** The expression of CD53 in MN group was higher than that of normal control. **(E)** The expression of CTSS in MN group was higher than that of normal control **(F)** The expression of FCER1G in MN group was higher than that of normal control **(G)** The expression of NCF2 in MN group was higher than that of normal control. *p* < 0.05 was considered statistically significant. *p < 0.05, **p < 0.01, ***p < 0.001. MN: membranous nephropathy; mRNA: messenger RNA.

**FIGURE 6:**
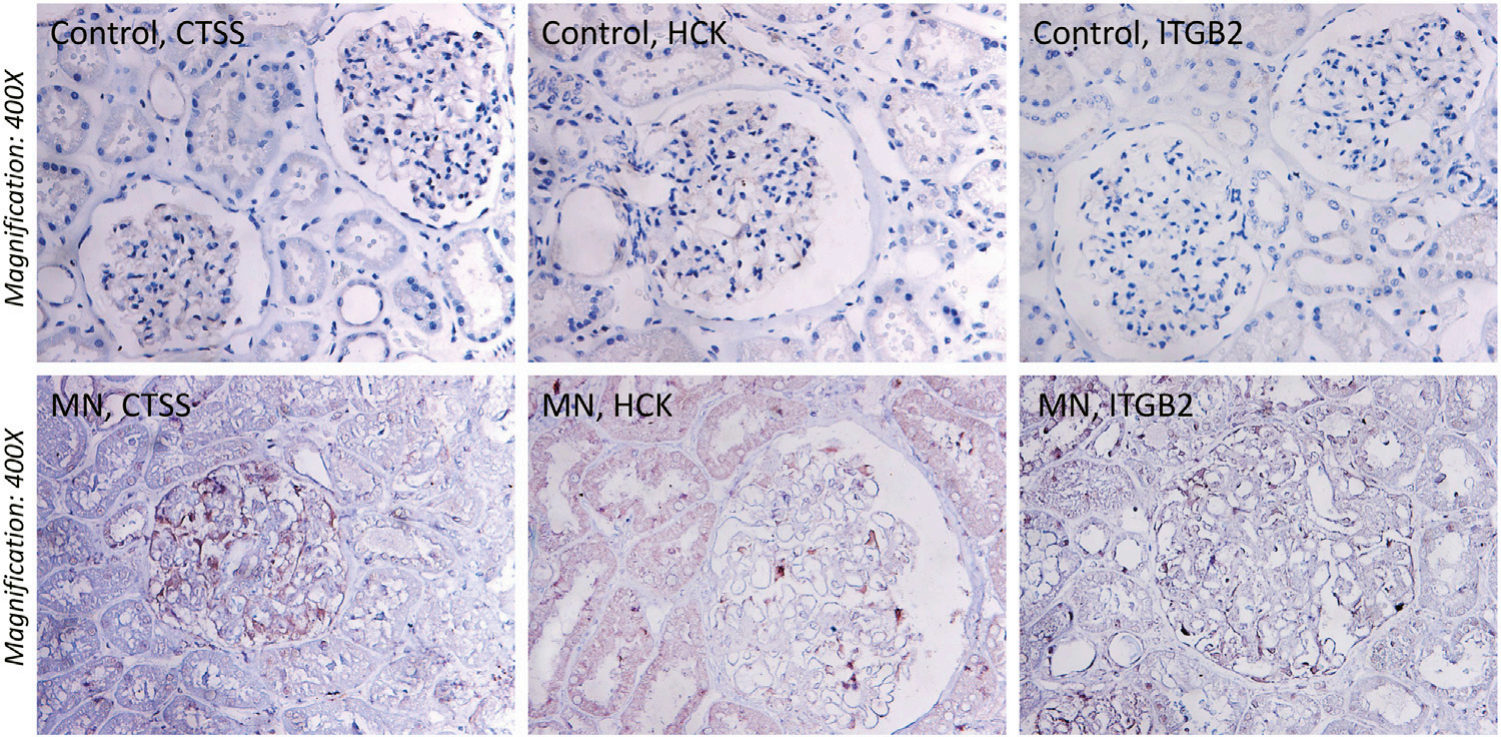
Immunohistochemistry (IHC) staining of CTSS, HCK, and ITGB2 in MN tissues and normal renal tissues. The expression of CTSS, HCK, and ITGB2 protein in MN tissues was higher than that in control group.

To ascertain the function of hub gene in glomerular lesion in MN, Nephroseq V5 online tool was used to perform subgroup analysis of hub gene. The results showed that hub gene (TYROBP, ITGB2, HCK, CD53, CTSS, FCER1G, and NCF2) expression differed between MN and healthy living donors (Figure 5); we found that 7 hub genes in MN kidney tissue was significantly higher than that of healthy kidney samples. We further examined the expression of ITGB2, HCK, and CTSS in MN or control tissues by IHC. As shown in Figure 6, ITGB2, HCK, and CTSS was up-regulated in glomerular region of MN patients, which showed brownish patchy or granular staining when compared to the control group.

## Discussion

A large proportion of patients with MN develop end-stage renal disease. MN is characterized by glomerular basement membrane thickening and podocyte injury, and disease progression is closely associated with inflammatory immune processes. Despite substantial research, the molecular pathways driving glomerular damage in MN remain unclear. We synthesized high-throughput microarray data and carried out comprehensive bioinformatics analysis to retrieve the information contained therein. Novel hub genes were found, giving trustworthy directions and insights for future investigation of the pathophysiology and related biomarkers of MN.

The KEGG enrichment analysis of GSEA and DEGs together suggested that MN group was positively associated with inflammation activation, chemokine expression, recruitment of various immune cells, complement and coagulation cascade pathways, suggesting that immune response processes involving antigen presentation and complement activation was crucial in MN pathogenesis. Mezzano et al. (2020) demonstrated that chemokines MCP-1, RANTES, and OPN were overexpressed in patients with progressive IMN and were relevant to monocyte recruitment, leading to kidney injury The number of mast cells has been reported to correlate with the progression of renal fibrosis in glomerulonephritis patients containing MN (Hiromura et al., 1998; Roberts and Brenchley, 2000). Meanwhile, interstitial monocytes/macrophages may contribute to the progression of MN through IgG subclass/complement deposition (Hu et al., 2021). Additionally, according to research done by Penny et al. (1998), CD8 (+) cytotoxic T lymphocytes were critical in mediating Heymann nephritis (HN) glomerular damage and may be relevant to the pathophysiology and therapy of MN. Much evidence demonstrated that, as a response to antibody-complement-mediated injury, podocytes underwent lysis, apoptosis, or activation, leading to glomerular injury in MN (Pippin et al., 2003; Nangaku et al., 2005). Confirming *in vivo* and *vitro* studies, C5a/C5aR1-mediated upregulation of local inflammatory responses and impaired phagocytosis led to chronic nephritis and renal fibrosis (Choudhry et al., 2016). Together, the above literature suggested that various types of immune molecules or immune cells and the complement system play an integral role in MN pathogenesis.

KEGG analysis also suggested that intercellular adhesion and aberrant expression of adhesion molecules might be crucially implicated in the MN etiology. In MN, substantial changes in the expression levels and distribution of vinculin were observed, which is a cytoplasmic protein that binds actin filaments to integrin-mediated cell-matrix adhesion and cadherin-based intercellular junctions (Lausecker et al., 2018). In addition, Disintegrin and Metalloproteinase 10 (ADAM10) in MN, a protease, is upregulated in inflammatory environment and mediates the shedding of extracellular domain of injury-associated cadherins leading to podocyte damage (Sachs et al., 2021). Also, serum containing PLA2R antibodies inhibited podocytes’ capacity to adhere to type IV collagen *in vitro*, providing evidence that serum soluble pathogenic factors disrupt podocyte adhesion in MN (Skoberne et al., 2014). The above molecules are associated with aberrant cell adhesion and thus are directly or indirectly involved in MN pathological processes.

GO-CC annotation of DEGs revealed that extracellular exosome, integral component of plasma membrane, extracellular space/matrix were predominantly enriched. Numerous structural components of the extracellular matrix were involved in the pathogenesis of MN. In MN, the thickening of the glomerular basement membrane was concomitant with increased spike formation containing extracellular matrix (ECM) proteins such as laminin, s-laminin, fibronectin, entactin, and acetyl heparan sulfate (Floege et al., 1992). The production and tissue deposition of basement membrane type IV collagen chain isoforms and fibrillar interstitial type I collagen were increased during immune injury in PHN animal model (Minto et al., 1998). Combined with the above proposed association of MN with abnormal adhesion, it is reasonable to hypothesize that anomalous alterations in structural components of the extracellular matrix are probably involved in the pathogenesis of MN by afflicting intercellular adhesion.

Both DEGs and GSEA GO-BP analysis showed major enrichment in pathways such as regulation of endothelial cell migration and sprouting angiogenesis in the MN group. Masuda et al. (2001) demonstrated that vascular endothelial growth factor (VEGF) potentiated glomerular capillary repair and expedited the recovery of experimentally induced glomerulonephritis, while VEGF downregulation resulted in the inability for angiogenic mechanisms to promote glomerular vascular repair in MN (Sivridis et al., 2003). Furthermore, increased expression of TGF beta receptor was associated with glomerular epithelial cell injury in experimental MN model (Shankland et al., 1996). In IMN, as an intrinsic endothelial cell antigen, expression of platelet endothelial cell adhesion molecule-1 (PECAM-1) in glomerular capillary is complete or partial absence, suggesting that angiogenesis mechanism of MN may be defective (Sivridis et al., 2003). These studies demonstrate that the progression of MN is inextricably linked to derangement of glomerular vascular repair mechanisms.

A total of 10 DEGs were identified as hub genes, including ITGB2, HCK, CTSS, TYROBP, CSF2RB, LAPTM5, FCER1G, NCF2, CYBB, and CD53, whose aberrant expression may contribute positively to the lesion progression and immunopathology of MN patients. Integrin beta 2 (ITGB2) is one of the subunits of integrins, a family of cell surface glycoproteins, which played a crucial role in leukocyte adhesion, migration and immune function (Hirahashi et al., 2009). Tissue analysis of kidney in rat model of nephrotic syndrome (NS) revealed increased expression of ITGB2 (CD18) in cytotoxic T lymphocytes, NK cells and monocytes (Pereira et al., 2015). ITGB2 (CD18) reacted with iC3b as a part of the leukocyte integrins CR3 (CD11b/CD18) and CR4 (CD11c/CD18), a critical mechanism by which complement promotes inflammation (Lachmann, 2009). Simultaneously, ITGB2 overexpression enhanced pro-inflammatory mediators in kidney, further deteriorating microvascular perfusion and histopathology, and decreasing renal function (Dehnadi et al., 2017), indicating that ITGB2 may contribute to disease progression in MN through complement system. As a member of the highly conserved Src family of cytoplasmic protein tyrosine kinases, hematopoietic cell kinase (HCK) transmits a variety of extracellular signals and affects cell proliferation and migration Wei et al. (2017) suggested that HCK was a key mediator for renal fibrosis, with the reason that HCK overexpression activated the transforming growth factor-β/Smad3 pathway *in vitro* experiments, which promoted fibroblast proliferation and inflammatory expansion. As a cysteine protease with intracellular and extracellular protein hydrolytic activity, Cathepsin-S (CTSS) can be transferred to the cell surface and released into the extracellular environment to participate in the destruction of extracellular matrix proteins. (Wilkinson et al., 2015). CTSS increases microvascular permeability and leukocyte adhesion during inflammation by activating protease-activated receptor (PAR)-2 on endothelial cells, leading to endothelial dysfunction (Steubl et al., 2017). Yao et al. have confirmed that CTSS mediated the regulation of renal fibrosis through the transforming growth factor-β/SMAD signaling pathway (Yao et al., 2019). As GFR decreased, CTSS and markers of endothelial dysfunction associated with inflammation, such as soluble tumor-necrosis-factor receptors (sTNFR) 1 and 2, increased (Steubl et al., 2017) and might be crucial to MN pathogenesis. Furthermore, elevated CTSS levels were related with activation of sTNFR1/2 in ESRD (Carlsson et al., 2015). These evidences indicate that CTSS may be a potential marker in MN progression to end-stage renal disease. Taken together, these findings will provide novel potential targets for future MN research.

Our study has several limitations. First, this study had a relatively small sample size and all data were obtained from online database. Further research with larger sample sizes and *in vitro*/*vivo* investigations are required to determine if the identified hub genes may be employed as diagnostic indicators or therapeutic targets for MN. Alternatively, it is better to do integrating analysis using bulk RNA-Seq data together with single cell RNA-Seq data of MN to get more accurate results. Second, the absence of specific clinical data in the GEO database makes it challenging to establish a more credible relationship between hub genes and different disease stages and future study could focus on specific type of MN, especially in idiopathic MN. Third, differentially expressed genes obtained using only healthy kidney tissue as control might yield specificity concerns, and future work could concretely focus on analyzing the potential discrepancies between MN and other primary nephropathy.

In this study, we applied gene microarray and bioinformatics techniques to evaluate the putative molecular processes and regulatory targets of MN. A total of 370 DEGs and 10 hub genes were screened, including ITGB2, HCK, CTSS, LAPTM5, CD53, etc. The abnormal expression of these genes is closely connected with different immunopathological processes, which might provide a better understanding for improving molecularly targeted therapeutic strategies of MN. Taken together, the above findings provide possible guidance to further investigate the pathological mechanisms of MN.

## Data Availability

The datasets presented in this study can be found in online repositories. The names of the repository/repositories and accession number(s) can be found in the article/supplementary material.
